# Phylogenetic Relationships of Immune Function and Oxidative Physiology With Sexual Selection and Parental Effort in Male and Female Birds

**DOI:** 10.1002/ece3.71119

**Published:** 2025-03-18

**Authors:** Péter L. Pap, Csongor I. Vágási, Veronika Bókony, Janka Pénzes, Krisztián Szabó, Nóra M. Magonyi, Gábor Á. Czirják, Orsolya Vincze

**Affiliations:** ^1^ Evolutionary Ecology Group, Hungarian Department of Biology and Ecology, Centre for Systems Biology, Biodiversity and Bioresources Babeş‐Bolyai University Cluj‐Napoca Romania; ^2^ Behavioural Ecology Research Group, Department of Evolutionary Zoology and Human Biology University of Debrecen Debrecen Hungary; ^3^ Department of Evolutionary Ecology, Plant Protection Institute HUN‐REN Centre for Agricultural Research Budapest Hungary; ^4^ Department of Zoology University of Veterinary Medicine Budapest Budapest Hungary; ^5^ Doctoral School of Biology and Sportbiology, Faculty of Sciences University of Pécs Pécs Hungary; ^6^ Department of Wildlife Diseases Leibniz Institute for Zoo and Wildlife Research Berlin Germany; ^7^ Institute of Aquatic Ecology, HUN‐REN Centre for Ecological Research Debrecen Hungary

**Keywords:** antioxidant, innate immunity, leukocyte, life history, oxidative damage, sex roles

## Abstract

Sexual differences in physiology are widely regarded as potential proximate mechanisms that underlie sex differences in mortality, life history and disease risk of vertebrates. However, little is known about the causes of sex‐specific variation in physiology. Sexual selection and parental workload are two key components suggested to play a role. Theory predicts that, within males, species with stronger male sexual selection (greater sexual dichromatism and more frequent social polygyny) and higher male parental effort should have lower immune capacity and stronger oxidative imbalance. Within females, a weak or no direct effect of male sexual selection on physiology is expected, but species where females invest more in parental care should have lower immune capacity and higher oxidative imbalance. We tested these predictions by phylogenetic comparative analyses conducted separately for the two sexes and based on 11,586 physiological measurements of samples collected in the field from 2048 individuals of 116 and 106 European bird species for males and females, respectively. For males, we found that the degree of dichromatism, polygyny and male parental effort correlated negatively with multiple immune indices, and the level of antioxidant glutathione correlated positively with polygyny score. In contrast, female immune and oxidative variables were unrelated or weakly related to both male sexual selection and female parental effort. We conclude that sex roles can drive inter‐specific variation in immune function (primarily in male birds), but less so in oxidative physiology. These findings support earlier claims that males pay higher physiological costs of sexual selection than females, but apparently also of caregiving. We discuss how females might avoid such costs.

## Graphical abstract

Our comparative analyses explored across‐species variation in physiology to evaluate the role of sexual selection and parental care in the evolution of immune and oxidative physiology. Our results highlight that male sexual selection and parental effort are powerful factors shaping male but not female immunity, but have no effect on oxidative physiology in either sex.

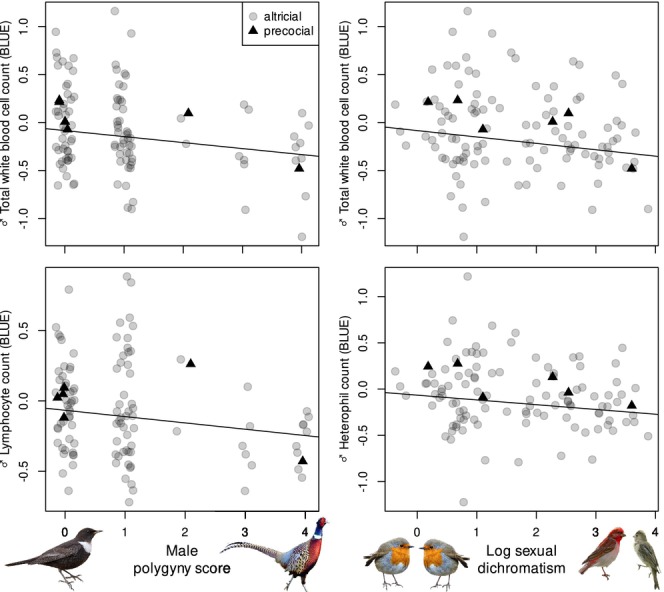

## 1 Introduction

Sex differences in physiology are widespread across animals, including humans (Nunn et al. 2009; Kelly et al. 2018; Vincze et al. 2022). Understanding such physiological sex differences is crucial as it is believed to be associated with sex differences in a set of life history, behavioural and epidemiological characteristics, including lifespan, infection rate and demography (Klein and Flanagan 2016). However, what factors cause sex differences in physiology is little understood. Sexual selection and investment in parental care might differ among sexes, leading to distinct sex roles with one sex (generally males) mostly competing for mates, while the other (generally females) provides care for the young (Andersson 1994). Sex roles were suggested to act as drivers of physiological sex differences across animals (Stoehr and Kokko 2006; Nunn et al. 2009; Vincze et al. 2022). However, it is still debated whether the difference in the strength of sexual selection and parental care is enough to produce a sex bias in physiology (Stoehr and Kokko 2006; Nunn et al. 2009; Vincze et al. 2022). Two across‐species studies based on large numbers of mammalian and avian species, respectively, found no correlation between the degree of sex differences in physiological traits and the strength of sexual selection or sex differences in parental care (Nunn et al. 2009; Vincze et al. 2022). These findings challenge the idea that sexual selection may be a key mediator of among‐species variation in physiological sex differences. This contradiction may be resolved by two considerations. First, strong sexual selection is unlikely to solely affect the competing sex (usually males in birds) engaged in sexual displays, aggressive interactions and territory defence. It may also indirectly impact the sex that provides more parental care (usually females in birds) because the burden of parental duties may increase in this sex due to the lower parental investment by their mates (Gonzalez‐Voyer et al. 2022). Thus, strong sexual selection in males and associated high parental effort in females might diminish sex differences in physiology because males pay the price of sexual selection, whereas females pay the price of increased maternal workload. Second, female physiology might evolve as an indirect consequence of selection on males due to correlated evolution (Møller et al. 2005). Correlated evolution arises because males and females of the same species share the majority of their genes; hence, selection on a particular trait in one sex might affect the same trait in the other sex as well (Cox and Calsbeek 2009). Under this scenario, we expect little influence of the intensity of sexual selection on the degree of physiological sex differences across species. Nonetheless, in both scenarios, we expect the effect of sexual selection and parental duties to manifest across species when considering sexes separately (see Table 1).

**TABLE 1:** Questions and predictions tested in the present study and in (Vincze et al. 2022). Across‐species correlations are denoted by the ~ sign; sex differences are indicated as ♂ – ♀.

Question	Prediction tested in the present study	Prediction tested in (Vincze et al. 2022)
Does the strength of sexual selection increase the intensity of physiological costs?	♂ physiology ~ ♂ sexual selection	Sexual differences in physiology ~ strength of sexual selection
Is parental care physiologically costly?	♂ physiology ~ ♂ parental care	Sex differences in physiology ~ sex differences in parental care
	♀ physiology ~ ♀ parental care	
Does sexual selection indirectly affect physiology via genetic correlation between sexes?	♀ physiology ~ ♂ sexual selection	Not relevant (no testable prediction for sex differences)

The effects of sexual selection on physiology can be manifold, potentially being mediated by energetic costs of sexual display, fight, resource defence and ornament production or by the pleiotropic effects of sex or stress hormones (Monaghan et al. 2009; Zuk 2009; Costantini 2014). Consequently, males of species under strong sexual selection are expected to be in a weaker physiological state (e.g., lower immune function and/or higher oxidative imbalance) due to their higher energetic or physiological stress (von Schantz et al. 1999; Garamszegi et al. 2005; Knowles et al. 2009; Foo et al. 2017). Similarly, high investment in parental activities has the potential to drain resources necessary for immune function and for the mitigation of oxidative stress (Knowles et al. 2009; Costantini 2014). For example, across species with similar intensity of male sexual selection, males of species with higher male parental effort are expected to suffer reduced immune function and higher oxidative stress as compared with males of species with less paternal effort.

Individual differences in sexual ornaments and parental workload within species were repeatedly shown to correlate with immune function and oxidative physiology across males or females separately (Knowles et al. 2009; Monaghan et al. 2009; Simons et al. 2012; Costantini 2014, 2018; Speakman and Garratt 2014; Blount et al. 2016). However, whether these factors can explain male–male or female–female variations across species has not yet been explored. Analysing sexes separately would permit testing the importance of sex roles (metrics of sexual selection and parental effort) in shaping physiology while looking beyond pure sex differences. Here we employ this sex‐specific modelling approach to test if across‐species variation in immune function and oxidative physiology measured during the breeding season is explained by sexual selection and parental workload separately in male and female birds. Comparing these results with those reported by Vincze et al. (2022) allows inferring whether the physiological consequences of sex roles manifest across species, unlike in the case of sex differences in physiology (see Table 1).

We measure the intensity of male sexual selection by social mating systems (i.e., degree of polygyny) as well as plumage dichromatism, representing genetic mating systems reflected by its correlation with extra‐pair paternity (Dunn et al. 2001; Liker et al. 2015). We predict lower immune indices and stronger oxidative stress in males of species subject to stronger sexual selection and exhibiting higher paternal workload. Similarly, we predict lower immune indices and stronger oxidative stress in females of species subject to more maternal workload. We did not test the effect of female sexual selection on female physiology because our sample only includes species with traditional (non‐reversed) sex roles where females typically engage in little, if any, intra‐ or inter‐sexual competition. However, we tested the relationship between female physiology and male sexual selection, predicting no relationship among these variables if only males suffer the costs of sexual selection. The alternative hypothesis predicts lower immune indices and stronger oxidative stress in females of species with stronger male sexual selection in case of indirect effects or correlated evolution between sexes.

## 2 Materials and Methods

### 2.1 Sample Collection

We use a large data set of 121 European species (116 species for males and 106 species for females), spanning across the avian phylogeny, representing 44 families and 14 orders. The data set comprises 11, physiological measurements of 2048 wild birds captured at various sites across Romania, between 2009 and 2013 and between 2016 and 2019. Sampling was restricted to the breeding season between April and July (when the effect of sex roles should be the most prominent), to the same geographic region, and was performed using identical field protocols (minimising handling stress) and uniform laboratory measurements (Vincze et al. 2022). We determined the sex of each individual in the field whenever this was possible based on morphological characteristics. We used molecular sexing in cases of sexually monomorphic species (for details, see Vincze et al. 2022). The data set is similar to Vincze et al. (2022), although the species pool and the sample size are larger in the present study. For the analyses in Vincze et al. (2022), we computed sex differences in physiological parameters and used the degree of difference as dependent variables. In the present analyses, we used the data of males and females in separate models; therefore, in the models, we could include the data of those species for which we could only obtain samples from one sex.

### 2.2 Physiological Variables

We detailed the protocols of laboratory assays in Vincze et al. (2022) and Marton et al. (2022). Immunity was quantified by counting the number of total and specific (heterophils, lymphocytes) white blood cells (WBCs) and expressed as approximately 10^4^ erythrocytes. We also computed the heterophil/lymphocyte (H:L) ratio. Rare leukocytes (basophils, monocytes and eosinophils) were omitted from the analyses due to their rarity in blood smears and the difficulty in precisely assessing across‐species variability. The total number of WBCs is a general measure of the amount of investment in immune defence (Semple et al. 2002; Nunn 2002; Nunn et al. 2003; Blount et al. 2003). Leukocytosis is most apparent in the case of abundant white blood cell types, while a differential increase in specific white blood cell types for a given body size can signal the underlying condition. For instance, an increase in heterophils marks upregulated inflammatory responses, whereas an increase in lymphocyte counts is indicative of a better general pathogen defence (Davis et al. 2008). Similarly, decreased levels of leukocytes may signal energetic and stress‐induced immunosuppression or reduced ability to fight parasites (Blount et al. 2003; Davis et al. 2008; Pap et al. 2015). The ratio of heterophils to lymphocytes is also considered a reliable proxy of physiological stress (Davis et al. 2008). Besides leukocytes, we also measured agglutination and lysis, which quantify the natural antibodies and complement levels, and we measured the bacteria killing activity (BKA) of the plasma against *Escherichia coli* . Natural antibodies and the activity of the complement system are two associated measures of the constitutive innate immune system (Matson et al. 2005). Higher scores mean that the immune constituents of the plasma can agglutinate or lyse foreign erythrocytes at lower concentrations (i.e., indicate better immune capacity). The bacteria killing assay is a direct measure of constitutive innate immunity in birds (Tieleman et al. 2005) and characterises the capacity of the blood components to limit bacterial infection. Reduced scores of both natural antibodies and bacteria killing assays may signal immunosuppression (Stier et al. 2009; Merrill et al. 2012).

Oxidative state was assessed by measuring the levels of two non‐enzymatic antioxidant markers (total antioxidant status, TAS and uric acid, UA) in the blood plasma and one non‐enzymatic antioxidant in erythrocytes (total glutathione, tGSH) and by the level of peroxidative damage to membrane lipids (malondialdehyde, MDA) and the levels of hydroperoxides produced by the oxidative damage of biomolecules (reactive oxygen metabolites, ROMs) measured in the plasma (detailed protocols can be found in Vágási et al. 2019, Marton et al. 2022). All oxidative physiology markers measured have previously been shown to be associated with fitness parameters in free‐living organisms. For instance, decreased non‐enzymatic antioxidant levels are associated with stress, increased reproductive effort and sexual activity (Alonso‐Alvarez et al. 2004; Wiersma et al. 2004; Metcalfe and Alonso‐Alvarez 2010; Vágási et al. 2019), although the change in UA concentration should be treated with caution because this product results from protein catabolism and varies with energy use (Alan and McWilliams 2013). Similarly, oxidative damage to lipids (i.e., MDA) and hydroperoxides produced by the oxidative damage of biomolecules (i.e., ROMs) are associated with higher reproductive effort and may increase in species under intense sexual selection (Monaghan et al. 2009; Dowling and Simmons 2009; Metcalfe and Alonso‐Alvarez 2010; Blount et al. 2016).

### 2.3 Predictor Variables

Male polygyny was scored based on information obtained from birdsoftheworld.org, Snow et al. (1998) and earlier studies (Olson et al. 2008; Liker et al. 2015) and following the methodology described in Olson et al. (2008) and Liker et al. (2015). In short, we scored the overall incidence of social polygyny on a scale from 0 to 4, with 0 corresponding to no (or very rare) polygyny (< 0.1% of individuals), 1 to rare polygyny (0.1%–1%), 2 to uncommon polygyny (1%–5%), 3 to moderate polygyny (5%–20%) and 4 to common polygyny (> 20%). Our scoring of polygamy was highly repeatable with those of Liker et al. (2015), as shown in Vincze et al. (2022). To quantify sexual dichromatism, plumage coloration was measured in both sexes on digital images of adults from illustrations of the Handbook of the Birds of the World Alive (del Hoyo et al. 2017) following the methodology described in Dale et al. (2015) and Carballo et al. (2020) and as used in Vincze et al. (2022). This measure of sexual dichromatism correlates positively with the rates of extra‐pair paternity (EPP) (Spearman's *r* = 0.33, *p* = 0.015, *n =* 55), which we measured as the percentage of extra‐pair offspring (available for 55 species out of the 124 species in our physiology data set) taken from Brouwer and Griffith (2019). In our multispecies database, dichromatism is mainly due to the colour variation of males, whereas the colour of the females varies little compared to the males. Using only the plumage colouration of females in the analysis would be misleading because females' colouration varies in parallel with male colouration, so in this case, we would be measuring the role of male sexual selection on females' physiology.

To characterise parental workload across species within each sex, we gathered information from Liker et al. (2015), birdsoftheworld.org or Snow et al. (1998) on six components of parental care following the methodology described in Liker et al. (2015): nest building, incubation, nest guarding (before hatching), chick brooding, chick feeding and chick guarding (after hatching). Each component was scored as follows: −1: only female care, −0.5: 1%–66% female care, 0: 34%–66% male care, 0.5: 67%–99% male care, 1: only male care. These scores were based on quantitative data if these were available (e.g., percentage of incubation by males) or on qualitative descriptions of care in the data source. Scoring was necessary because quantitative data were unavailable for many species (Liker et al. 2015). Overall pre‐ and post‐hatch care bias was then calculated as simple averages of the pre‐ and post‐hatch care components, respectively, for which data could be retrieved from literature. The scores obtained from Liker et al. (2015) correlate strongly with our scores (*r* = 0.87, *p* < 0.0001, *n* = 18). From these scores, we derived the percentage of pre‐hatching and post‐hatching care provided by each sex (i.e., –1 equalling 100% female, 1 equalling 100% male care). Then we calculated parental effort as clutch size × [(length of incubation × % pre‐hatching care) + (length of chick rearing × % post‐hatching care)]; that is the relative contribution of each sex was multiplied by the length of each phase of parental care and the number of offspring in the nest. Data on clutch size and the length of incubation and chick rearing were retrieved from Storchová and Hořák (2018).

Given that across‐species variation in physiology is strongly dependent on body size (Pap et al. 2015), we aimed to control for the effect of body mass in each model. Data on sex‐specific body mass was extracted from Storchová and Hořák (2018) but were verified using alternative sources, and some data were updated using recent primary literature data or our field measurements. Note that including body mass as a covariate also controls for the effect of body size on the length of parental care, that is comparisons of care scores refer to the same body size. We also extracted information on the diet of each species because our former analyses indicate that measures of oxidative physiology depend on food type (Marton et al. 2022), so we included this variable to control for diet‐related variation in the physiological data. Diet was extracted from Storchová and Hořák (2018) as a categorical variable with three levels: animal (mostly animal‐based, including carnivores and insectivores), plant (mostly plant‐based) or omnivorous (~50%–50% consumption of animal and plant food). Data on body mass, diet and measures of sexual selection will be available in the Dryad repository.

### 2.4 Statistical Analyses

All analyses were run in the R computing environment version 4.0.4 (R Development Core Team 2022). Despite standardised sampling and laboratory processing of samples, differences in physiological parameters could be detected across years of sample collection (Vincze et al. 2022). To control for this potentially confounding effect, we extracted species‐ and sex‐specific average physiology measures as best linear unbiased estimates (BLUEs). To do so, we constructed general or generalised (with Poisson error distribution in case of agglutination and lysis and binomial error distribution for BKA) linear mixed models (using R function *lmer*, package *lme4*; Bates et al. 2015). All models contained a physiological parameter as a dependent variable and two random factors: year and the combination of species and sex. Poisson and binomial models included a third, observation‐level random factor to allow for overdispersion. BLUEs were then extracted from these models as the conditional means (from linear models) or conditional modes (from Poisson and binomial models) for each species‐sex combination (using R function *ranef*, package *lme4*). To ensure model fit, MDA, UA, TAS, tGSH and ROMs were square‐root transformed; WBC, heterophil and lymphocyte counts were natural log(*x* + 1) transformed, while H:L ratio was natural log(*x* + 0.1) transformed prior to BLUE extractions. Transformations were selected based on the scale of the variables and based on the distribution of residuals of models used to extract the BLUEs. All log transformations in the analyses and figures are natural logarithms. H:L ratios could not always be calculated due to zero lymphocyte counts in some individuals; therefore, sample sizes vary slightly for different white blood cell parameters. Sample sizes and BLUEs for each measured physiological parameter will be available in the Dryad repository.

Subsequently, phylogenetic generalised least squares (PGLS) models were constructed (using R function *gls* from package *nlme*; Pinheiro et al. 2021) for each physiological parameter, separately for males and females, using BLUEs as dependent variables. For the males models, we included the following predictors: polygyny score, sexual dichromatism, male parental effort, male body mass and diet. Similarly, for female's models we used the following predictors: polygyny score, sexual dichromatism, female parental effort, female body mass and diet. Weighting models by sample size of each species did not improve model fit for any of the measured physiological parameters in either males or females; therefore, these weights were omitted from all presented models. Due to detected heteroscedasticity of the residuals in relation to polygyny scores, we included a variance structure in all models, using the *varIdent* function to allow for differences in variance between species with low (0 or 1) vs. high (2–4) polygyny scores. To control for the non‐independence of species‐specific data points, all models included a phylogenetic covariance structure. To do this, we obtained 1000 equally parsimonious phylogenies from http://birdtree.org (Jetz et al. 2012) using the Hackett backbone tree (Hackett et al. 2008), and we assembled a rooted, ultrametric consensus tree using the *consensus.edges* function of the *phytools* R package (Revell 2012). This consensus tree was then used in all presented PGLS models, and phylogenetic signal (Pagel's lambda, *λ*) was estimated by maximum likelihood approximation in each model; *λ* values that converged to negative estimates were fixed at zero. While our sample included species from a wide taxonomic range, the overwhelming majority of the sampled taxa were altricial (114 of the 121 species). Given that developmental mode is associated with marked differences in parental provisioning and because of the weak sampling of precocial species, we always tested the consistency of our results by repeating the analyses after excluding precocial species. For the latter, BLUEs were re‐extracted including only altricial species.

Multi‐collinearity did not affect our analyses, as shown by the following correlations between the predictor variables. Polygyny score and sexual dichromatism were not significantly correlated with each other (Spearman's *r* = 0.11, *p* = 0.2036, *n* = 121). Furthermore, polygyny was also not correlated with EPP (Spearman's *r* = 0.08, *p* = 0.5497, *n* = 55), corroborating that polygyny and sexual dichromatism reflect two independent components of sexual selection, that is, social and genetic mating systems. Female parental effort did not correlate with any measure of male sexual selection (*n* = 121; sexual dichromatism: Spearman's *r* = −0.12, *p* = 0.2057; polygyny score: Spearman's *r* = −0.03, *p* = 0.7592), whereas male parental effort was weakly negatively correlated with sexual selection indices (*n* = 124; sexual dichromatism: Spearman's *r* = −0.18, *p* = 0.0547; polygyny score: Spearman's *r* = −0.22, *p* = 0.0170). The latter weak correlations did not inflate our model estimates because the variance inflation factor was low (< 2) for both (i.e., male and female) sets of predictor variables.

## 3 Results

### 3.1 Immune Function in Males

**FIGURE 1:**
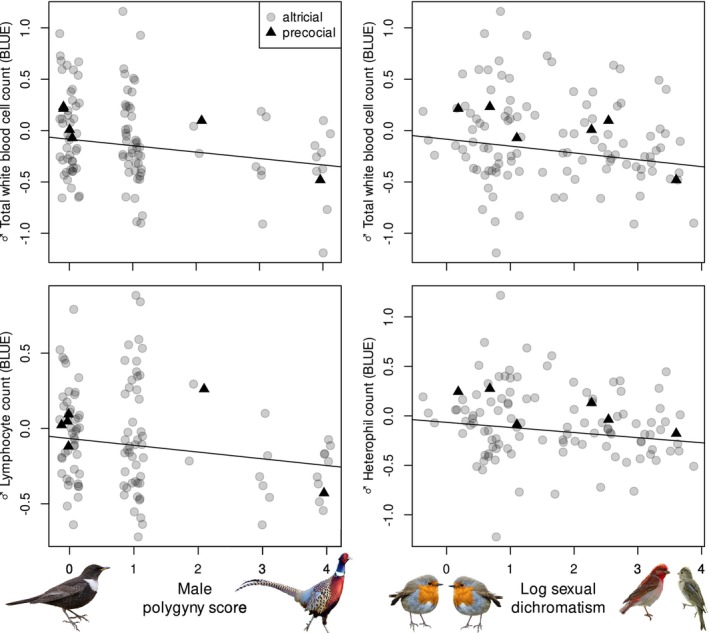
Associations between male white blood cell counts and measures of sexual selection (polygyny score and sexual dichromatism) across species. Higher scores indicate greater polygyny and dichromatism. Each point represents a species, with altricial and precocial species being marked with grey circles and black triangles, respectively. Fitted lines originate from models presented in Table 2.

**FIGURE 2:**
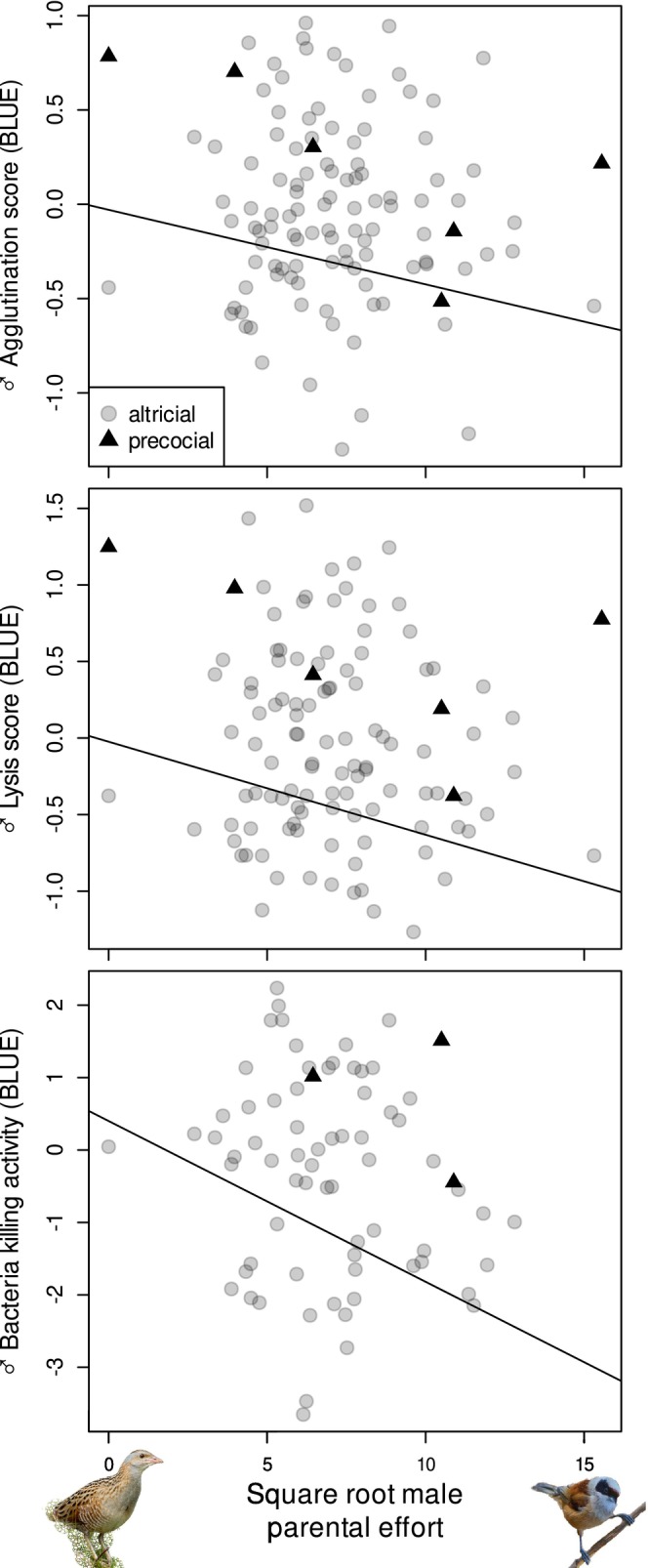
Associations between measures of male immune function (agglutination, lysis and bacteria killing activity) and male parental effort (higher scores indicating greater effort) across species. Each point represents a species, with altricial and precocial species being represented by grey circles and black triangles, respectively. Fitted lines originate from models presented in Table 2.

In males, polygyny score correlated negatively with WBC and lymphocyte counts (Table 2A, Figure 1). Sexual dichromatism was also negatively associated with WBC, heterophil and lymphocyte counts, although the latter relationship was marginally non‐significant (Table 2A, Figure 1). Male parental effort also had significant negative relationships with agglutination and lysis scores and with BKA (Table 2A, Figure 2). Males of species with plant‐based diets had lower agglutination and lysis scores and lower BKA than species with mainly animal‐based diets, with omnivores having intermediate values (Table 2A). Male body mass correlated positively with male immunological variables, excluding the H:L ratio (Table 2A). Excluding precocial species from the analyses did not affect the results qualitatively, except that the correlations of polygyny score with the number of WBC and lymphocytes were no longer significant (Table S1A).

**TABLE 2:** Phylogenetic generalised least square models explaining the variation in immune variables between species in relation to polygyny score, sexual dichromatism, parental effort, food type and body mass in males (A) and females (B). Each column represents a separate multivariate model with the variable indicated in the column heading as a response variable. In each cell, we report the slope (its standard error in brackets) on the top and *t*‐values from PGLS model summaries at the bottom of the cell. Statistical significance of each parameter is coded as *p < 0.05, **p < 0.01, ***p < 0.001 (highlighted in bold), while marginally significant *p*‐values (0.05 < *p* < 0.1) are marked with ^§^. The intercept refers to species with animal‐based diets and zero values for the other predictors; all other parameter estimates express slopes or differences from the intercept. Model statistics are reported in the last line of the table, including the strength of phylogenetic signal (Pagel's λ) and the degrees of freedom (d.f., overall and residual) for each model.

	White blood cell count	Heterophil count	Lymphocyte count	H:L ratio	Agglutination	Lysis	Bacteria killing activity
	*β* (SE)	*β* (SE)	*β* (SE)	*β* (SE)	*β* (SE)	*β* (SE)	*β* (SE)
	*t*‐value	*t*‐value	*t*‐value	*t*‐value	*t*‐value	*t*‐value	*t*‐value
**A. Male**							
Intercept	**−0.71 (0.15)** −4.75***	**−0.66 (0.12)** −5.61***	**−0.31 (0.13)** −2.33*	−0.13 (0.16) –0.84	−0.32 (0.24) −1.35	−0.42 (0.30) −1.38	0.40 (0.93) 0.43
Polygyny score	**−0.06 (0.03)** −2.31*	−0.03 (0.03) −1.17	**−0.06 (0.02)** −2.91**	0.00 (0.03) 0.07	−0.03 (0.03) −1.08	−0.00 (0.05) −0.04	−0.13 (0.14) −0.91
Sexual dichromatism	**−0.07 (0.03)** −2.27*	**−0.05 (0.02)** −2.24*	−0.05 (0.03) −1.72^§^	−0.01 (0.03) −0.35	0.05 (0.04) 1.26	0.03 (0.05) 0.61	0.10 (0.14) 0.74
Parental effort	0.01 (0.01) 0.68	0.00 (0.01) 0.11	0.00 (0.01) −0.04	0.00 (0.01) 0.01	**−0.04 (0.02)** −2.43*	**−0.06 (0.02)** −2.76**	**−0.22 (0.07)** −3.06**
Diet^a^							
Omnivorous	0.07 (0.09) 0.74	0.07 (0.07) 1.00	−0.04 (0.09) −0.46	0.12 (0.09) 1.32	−0.09 (0.12) −0.75	−0.07 (0.16) −0.47	−0.48 (0.45) −1.07
Herbivorous	−0.07 (0.12) −0.58	−0.08 (0.09) −0.89	−0.07 (0.12) −0.64	−0.00 (0.12) −0.04	**−0.57 (0.17)** −3.35**	**−0.72 (0.21)** −3.36**	**−1.83 (0.58)** −3.15**
Male body mass	**0.20 (0.03)** 6.71***	**0.19 (0.02)** 8.15***	**0.11 (0.03)** 4.07***	0.04 (0.03) 1.42	**0.16 (0.04)** 4.27***	**0.23 (0.05)** 4.52***	**0.34 (0.16)** 2.12*
Pagel's *λ* (d.f.)	0.00 (114, 107)	0.00 (114, 107)	0.00 (114, 107)	0.08 (112, 105)	0.28 (113, 106)	0.23 (113, 106)	0.43 (72, 65)
**B. Female**							
Intercept	**−0.50 (0.22)** −2.31*	**−0.43 (0.17)** −2.53*	−0.25 (0.20) −1.28	0.03 (0.13) 0.19	−0.47 (0.25) −1.87^§^	−0.34 (0.37) −0.93	0.76 (1.36) 0.56
Polygyny score	−0.02 (0.03) −0.45	−0.01 (0.03) −0.20	0.00 (0.03) 0.01	−0.02 (0.02) −1.21	0.02 (0.03) 0.49	0.04 (0.05) 0.88	−0.19 (0.16) −1.23
Sexual dichromatism	−0.03 (0.03) −0.86	−0.04 (0.03) −1.47	0.00 (0.03) −0.01	−0.04 (0.02) −1.77^§^	0.00 (0.04) 0.01	−0.06 (0.06) −1.09	−0.23 (0.17) −1.36
Parental effort	−0.02 (0.02) −1.06	−0.01 (0.01) −0.74	−0.01 (0.01) −0.94	0.00 (0.01) 0.08	−0.01 (0.02) −0.45	0.00 (0.03) −0.08	−0.05 (0.08) −0.64
Diet^b^							
Omnivorous	**−0.26 (0.11)** −2.29*	−0.16 (0.09) −1.81^§^	−0.20 (0.11) −1.87^0.07^	0.13 (0.09) 1.35	−0.07 (0.13) −0.54	0.13 (0.19) 0.69	0.84 (0.56) 1.51
Herbivorous	0.01 (0.12) 0.07	−0.02 (0.10) −0.20	0.00 (0.12) −0.02	0.13 (0.10) 1.34	0.03 (0.16) 0.19	−0.28 (0.24) −1.15	0.67 (0.62) 1.07
Female body mass	**0.24 (0.03)** 7.21***	**0.19 (0.03)** 7.22***	**0.14 (0.03)** 4.32***	0.01 (0.02) 0.42	**0.16 (0.04)** 4.05***	**0.16 (0.06)** 2.75**	0.14 (0.18) 0.77
Pagel's *λ* (d.f.)	0.00 (103, 98)	0.00 (103, 98)	0.00 (103, 98)	0.00 (97, 92)	0.20 (100, 95)	0.25 (100, 95)	0.65 (58, 53)

^a^ Contrasts between omnivorous and herbivorous groups (with standard errors in brackets): white blood cells 0.14 (0.14), *t* = 1.02, *p* = 0.5673, heterophils 0.16 (0.11), *t* = 1.47, *p* = 0.3105, lymphocytes 0.03 (0.13), *t* = 0.24, *p* = 0.9634, H:L ratio 0.12 (0.13), *t* = 0.93, *p* = 0.6244, agglutination **0.48 (0.19), *t* = 2.53, *p* = 0.0344**, lysis **0.64 (0.24), *t* = 2.69, *p* = 0.0252**, bacteria killing activity 1.35 (0.64), *t* = 2.10, *p* = 0.0985.

b Contrasts between omnivorous and herbivorous groups (with standard errors in brackets): white blood cells −0.26 (0.12), *t* = −1.70, *p* = 0.2108, heterophils −0.14 (0.13), *t* = −1.13, *p* = 0.4978, lymphocytes −0.20 (0.15), *t* = −1.32, *p* = 0.3913, H:L ratio − 0.01 (0.13), *t* = −0.01, *p* = 0.9982, agglutination −0.10 (0.20), *t* = −0.52, *p* = 0.8632, lysis 0.40 (0.28), *t* = 1.42, *p* = 0.3330, bacteria killing activity 0.17 (0.75), *t* = 0.23, *p* = 0.9706.

### 3.2 Immune Function in Females

Measures of male sexual selection and female parental effort were unrelated to all immune measures of females (Table 2B). Females of omnivorous species had a lower WBC count than females of species with animal‐based diets. Female body mass correlated positively with WBC, heterophil and lymphocyte counts, agglutination and lysis scores (Table 2B). Excluding precocial species from the analyses did not affect the results qualitatively, except in the case of the H:L ratio, which became significantly negatively correlated with sexual dichromatism (Table S1B).

### 3.3 Oxidative Physiology in Males

**FIGURE 3:**
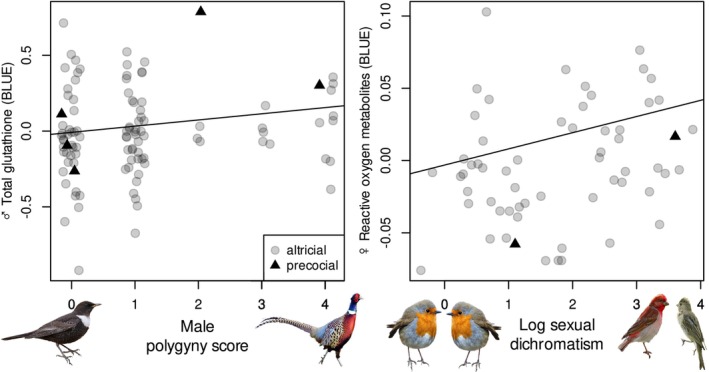
Association between male total glutathione level and polygyny score and female reactive oxygen metabolites and sexual dichromatism across species. Higher scores indicate greater polygyny and greater dichromatism. Each point represents a species, with altricial and precocial species being represented by grey circles and black triangles, respectively. Fitted line originates from model presented in Table 3.

The levels of tGSH increased significantly with polygyny score (Table 3A, Figure 3). Levels of TAS, UA, ROMs and MDA were unrelated to all tested measures of sexual selection or with male parental effort (Table 3A). Males of species with plant‐based and omnivorous diets had the lowest levels of UA and MDA but the highest ROMs, while males of species with animal‐based diets had the lowest tGSH levels, with omnivores having intermediate values (Table 3A). Male body mass correlated positively with tGSH and negatively with levels of ROMs (Table 3A). Excluding precocial species from the analyses only slightly affected the results, with the correlation between polygyny score and tGSH levels becoming non‐significant (Table S2A).

**TABLE 3:** Phylogenetic generalised least square models explaining the variation in oxidative physiology measures between species in relation to polygyny score, sexual dichromatism, parental effort, food type and body mass in males (A) and females (B). Each column represents a separate multivariate model with the variable indicated in the column heading as a response variable. In each cell, we report the slope (its standard error in brackets) on the top and *t*‐values from PGLS model summaries at the bottom of the cell. Statistical significance of each parameter is coded as *p < 0.05, **p < 0.01, ***p < 0.001 (highlighted in bold), while marginally significant *p*‐values (0.05 < *p* < 0.1) are marked with ^§^. The intercept refers to species with animal‐based diets and zero values for the other predictors; all other parameter estimates express slopes or differences from the intercept. Model statistics are reported in the last line of the table, including the strength of phylogenetic signal (Pagel's λ) and the degrees of freedom (d.f., overall and residual) for each model.

	Total antioxidant status	Uric acid	Total glutathione	Reactive oxygen metabolites	Malondialdehyde
	*β* (SE)	*β* (SE)	*β* (SE)	*β* (SE)	*β* (SE)
	*t*‐value	*t*‐value	*t*‐value	*t*‐value	*t*‐value
**A. Male**					
Intercept	0.02 (0.01), 1.79^§^	0.25 (0.18), 1.38	−0.34 (0.14), −2.52*	0.01 (0.03), 0.14	0.12 (0.07), 1.74^§^
Polygyny score	−0.00 (0.00), −0.52	0.04 (0.03), 1.59	0.04 (0.02), 2.15*	0.00 (0.01), 0.62	0.00 (0.01), 0.24
Sexual dichromatism	0.00 (0.00), 0.36	0.01 (0.04), 0.31	0.01 (0.02), 0.52	0.01 (0.01), 1.56	0.00 (0.01), 0.20
Parental effort	−0.00 (0.00), −1.58	−0.01 (0.02), −0.79	−0.00 (0.01), −0.21	0.01 (0.00), 1.54	−0.00 (0.01), −0.66
Diet^a^					
Omnivorous	−0.02 (0.01), −1.88^§^	−0.24 (0.13), −1.90^§^	0.14 (0.09), 1.59	0.04 (0.02), 2.25*	−0.11 (0.04), −2.61*
Herbivorous	0.00 (0.01), 0.10	−0.41 (0.16), −2.59*	0.22 (0.11), 2.04*	0.07 (0.03), 2.62*	−0.16 (0.05), −3.06**
Male body mass	−0.00 (0.00), −0.83	−0.04 (0.04), −1.10	0.08 (0.02), 3.14**	−0.02 (0.01), −2.91**	−0.02 (0.01), −1.35
Pagel's *λ* (d.f.)	0.00 (106, 99)	0.00 (107, 100)	0.14 (105, 98)	0.05 (66, 59)	0.16 (106, 99)
**B. Female**					
Intercept	0.02 (0.01), 1.89^§^	0.25 (0.22), 1.11	−0.09 (0.22), −0.42	−0.04 (0.02), −1.78	0.08 (0.08), 1.05
Polygyny score	0.00 (0.00), −1.51	−0.04 (0.03), −1.30	0.01 (0.02), 0.37	0.01 (0.00), 1.33	0.00 (0.01), −0.10
Sexual dichromatism	0.00 (0.00), −0.52	0.00 (0.03), 0.05	0.02 (0.03), 0.76	0.01 (0.00), 2.46*	0.00 (0.01), 0.11
Parental effort	−0.00 (0.00), −0.06	0.01 (0.02), 0.74	−0.02 (0.01), −1.96^§^	0.00 (0.00), 0.82	0.00 (0.01), 0.21
Diet^b^					
Omnivorous	−0.00 (0.01), −0.49	0.04 (0.13), 0.34	0.00 (0.09), −0.03	−0.01 (0.02), −0.41	−0.04 (0.04), −1.10
Herbivorous	−0.01 (0.01), −1.69^§^	−0.30 (0.15), −1.93^§^	0.18 (0.11), 1.68	−0.03 (0.02), −1.35	−0.14 (0.04), −3.19**
Female body mass	−0.00 (0.00), −2.22*	−0.09 (0.04), −2.57*	0.08 (0.03), 3.14**	0.00 (0.01), 0.34	−0.02 (0.01), −1.32
Pagel's *λ* (d.f.)	0.00 (91, 86)	0.21 (93, 88)	0.67 (94, 89)	0.15 (60, 55)	0.23 (94, 89)

^a^ Contrasts between omnivorous and herbivorous groups (with standard errors in brackets): total antioxidant status −0.02 (0.01), *t* = −1.48, *p* = 0.3093; uric acid 0.17 (0.18), *t* = 0.96, *p* = 0.6017; total glutathione −0.08 (0.12), *t* = −0.67, *p* = 0.7809; reactive oxygen metabolites −0.02 (0.03), *t* = −0.85, *p* = 0.6756; malondialdehyde 0.05 (0.06), *t* = 0.80, *p* = 0.7048.

^b^ Contrasts between omnivorous and herbivorous groups (with standard errors in brackets): total antioxidant status 0.01 (0.01), *t* = 1.01, *p* = 0.5685, uric acid 0.34 (0.19), *t* = 1.82, *p* = 0.1739, total glutathione −0.18 (0.13), *t* = −1.41, *p* = 0.3424, reactive oxygen metabolites 0.02 (0.02), *t* = 0.84, *p* = 0.6792, malondialdehyde 0.10 (0.06), *t* = 1.86, *p* = 0.1602.

### 3.4 Oxidative Physiology in Females

Measures of male sexual selection and female parental effort were unrelated to oxidative physiology variables in females, except for a significant positive correlation between sexual dichromatism and the level of ROMs (Table 3B, Figure 3). Female MDA level was significantly lower in species with plant‐based diets than in females of species with animal‐based diets (Table 3B). Female body mass correlated negatively with female TAS and UA levels and positively with tGSH levels (Table 3B). Excluding precocial species from the analyses did not affect the results qualitatively (Table S2B).

## 4 Discussion

Our comparative analyses of seven immunological and five oxidative physiological parameters of male and female birds yielded several insightful results. First, our analyses showed that two important measures of sexual selection, namely male social mating system (ranging from strict monogamy to common polygyny) and sexual dichromatism (a proxy of genetic mating system) were significantly negatively associated with several haematological traits of males across species. Males of species subject to strong sexual selection had lower counts of total white blood cells, heterophils and lymphocytes. These findings support the conclusions of former comparative studies on the variation in immune function of birds and mammals, measured for both sexes pooled together (Møller 1997; Nunn et al. 2000; Anderson et al. 2004). Second, our proxies of sexual selection had no or weak relationship with oxidative physiology in male‐ and female‐specific models alike. Across the five inspected oxidative physiology markers, only the total glutathione level increased significantly with polygyny score in male‐specific models. Third, we found weak evidence for indirect or correlated evolutionary effects of male sexual selection on female physiology, except for a positive correlation between sexual dichromatism and the concentration of the reactive oxygen metabolites in females. Fourth, contrary to our prediction, the physiological cost of parental workload explained among‐species variation in male immune function, but not that of females, while its importance was undetectable in the case of oxidative state in either sex. Our results are intriguing because they suggest that the reason Vincze et al. (2022) have not previously found a correlation in sex differences of immune measures and sex bias in measures of sexual selection and parental care is that males pay the price in immunosuppression whichever they invest in (sexual selection or parental effort), while the correlations in females are weak. The reasons for the different effects of sexual selection and parental effort on males and females are discussed below.

### 4.1 Sexual Selection and Physiology

Our comparative analyses revealed that, according to theoretical predictions, male immune indices are lower in species that experience stronger male sexual selection. A lower number of leukocytes for a given body size may indicate reduced immune activity in the blood, possibly due to elevated stress (Davis et al. 2008). In males, such an effect may potentially be induced by costly sexual behaviour and/or by sex hormones associated with male reproductive activities (von Schantz et al. 1999; Garamszegi et al. 2005; Knowles et al. 2009; Foo et al. 2017). Our findings support this explanation for immune function (Costantini 2014) and indicate an evolutionary trade‐off: species in which males engage in intense and costly sexual activity evolved lower investment in the number of leukocytes (Wedekind and Folstad 1994). Interestingly, two comparative studies on leukocyte counts and mating systems across mammalian species have shown an exactly opposite pattern, although they quantified physiology at the species level without considering the potential sex‐specificity of the relationships. These studies found higher white blood cell counts in species with males having a higher number of mates and argued that the association is likely the consequence of increased disease risk in species with multiple matings (Nunn et al. 2000, Anderson et al. 2004). The contrasting results of the latter two studies and our present findings indicate potentially different mechanisms driving the association between sexual selection and physiology in mammals and birds, which may not be surprising given the radically different male reproductive strategies in the two taxonomic classes. For instance, in mammals, social monogamy occurs in only ca. 9% of species with some form of male contribution to parental care in only ca. 5.4% (Lukas and Clutton‐Brock 2013). In contrast, 81% of birds form pair bonds and provide biparental care, with an additional 9% of species breeding cooperatively (Cockburn 2006). Nevertheless, it is worth posing the question of whether the results of these former comparative studies would change if physiological measurements of males and females from various seasons had not been indiscriminately pooled because the physiology of the two sexes can differ considerably (Vincze et al. 2022).

Although the negative effect of sexual selection should apply for oxidative physiology as well, knowing that stress exposure may cause oxidative imbalance (Costantini 2014), our approach does not support this prediction as we found no relationship between sexual selection and oxidative physiology of males. Only male total glutathione concentration was related to the polygyny score, and even this relationship was contrary to the predicted direction (positive association observed, negative association predicted). These results are somehow surprising, given the multitude of experimental studies showing the oxidative cost of sexual activity or sex hormones (von Schantz et al. 1999; Monaghan et al. 2009; Costantini 2014). Nevertheless, our results are in line with a recent study, where we found that sexes do not differ in four measures of oxidative physiology and that proxies of sexual selection were unrelated to sex differences in oxidative state (Vincze et al. 2022).

### 4.2 Physiological Cost of Parental Effort

Our analyses pointed out a consistent negative covariation between male parental effort and three important measures of male humoral immune function, namely agglutination, lysis and bacteria killing activity of the blood. These results strongly support the physiological cost of reproduction in terms of impaired immune function (Knowles et al. 2009). In contrast, we found no statistically significant correlation between maternal effort and any physiological parameters. This finding may be surprising because, across birds, females generally invest more in parental duties than males, and this sex bias in care applies to the majority of species in our data set (Dryad Table 2). Thus, we had expected the immune‐suppressive effect of parental workload to manifest across species, especially in females and not necessarily in males. One possible explanation for our results is that, during breeding, males are under physiological stress because of competition, territorial defence or mate attraction activities, which might make them more vulnerable to stress due to parental effort. Indeed, males have higher annual mortality rates than females in species in which sexual selection on males is more accentuated (Liker and Székely 2005). Another, non‐exclusive explanation can be that females might have evolved higher investment into those components of the immune system which are covered by our measures for allocating these into eggs in order to protect the developing embryos (Saino et al. 2001), and variation due to this maternal immune boosting might complicate the across‐species relationships that we studied here. A third alternative explanation could be that evolutionary trade‐offs involving physiological traits may manifest differently between males and females, resulting in different immune effectors being compromised in the two sexes (Klein and Flanagan 2016).

We found no support for the oxidative cost of parental effort, as none of the measures of the antioxidant system or oxidative damage co‐varied with parental effort in either males or females. Contrary to these results, in a meta‐analysis addressing the oxidative cost of reproduction in female birds and mammals, the oxidative damage in mothers measured across tissues and with different markers increased with reproductive effort (Blount et al. 2016). One explanation for the lack of correlation between parental care and oxidative physiology parameters may be that, as measures of oxidative physiology vary with breeding status (Pap et al. 2018), the noise induced by the sampling period does not allow the cost of breeding to be detected. Therefore, additional high‐resolution data sets are needed in which the breeding status of individuals is determined.

### 4.3 Sex‐Specific Evolution of Physiology

Female physiology may be driven by selection on males either via genetic correlation or through an indirect relationship, whereby strong sexual selection on males forces females to allocate more into parental care and pay the resulting physiological costs. However, our results do not support these scenarios for several reasons. First, we found no relationship between female physiological parameters and the indices of sexual selection in males. There was one exception: females had a higher load of reactive oxygen metabolites in species with higher sexual dichromatism. This might indicate higher oxidative stress in females when males allocate more into extra‐pair mating as reflected by their bright plumage; however, an alternative explanation is also possible. Sexual dichromatism is influenced not only by sexual selection on males but also by natural selection on females for cryptic coloration, and the latter may be stronger in species with higher maternal effort (Badyaev and Hill 2003; Jordan Price 2019). Therefore, this particular correlation that we found might simply reflect the direct effects of maternal effort on both physiology and coloration, rather than an indirect effect of male sexual selection. Second, the relationships we found in males between physiological parameters and the indices of sexual selection and parental care were not mirrored by similar relationships in females. In other words, no correlation that was significant in males was significant in females and *vice versa*. These results suggest that female physiology can evolve relatively independently of male physiology, facilitating the evolution of sex‐specific optimisation (Metcalf and Graham 2018).

A caveat that has to be borne in mind is that we did not formally compare the strength of relationships between the sexes, as our aim was to test predictions formulated for each sex separately (Table 1). The required data structure and analytical framework are different for these two goals of comparing sexes on one hand and conducting sex‐specific analyses on the other hand. Therefore, it remains possible that some of the tested relationships have similar strength in both sexes, but the slightly larger sample size for males yielded higher statistical power for detecting the same relationship as significant. However, we consider this unlikely, as the difference in sample size is small (10 species), while the differences in effect size are relatively large in cases where males, but not females, show a significant correlation with polygyny, sexual dichromatism, or parental care (see *t*‐values in Tables 2 and 3). Therefore, we can conclude that male immunity covaries with both paternal care and sexual selection across species, while for females the analogous relationships are either weaker or are burdened by more noise, possibly due to the reasons discussed above.

## 5 Conclusions

Our comparative analyses explored across‐species variation in physiology to evaluate the role of sexual selection and parental care in the evolution of immune and oxidative physiology. We used a new approach by analysing male and female birds separately to reconcile a conflict between earlier results on this matter and theoretical predictions. Our results highlight that male sexual selection and parental effort are powerful factors shaping male but not female immunity, nor oxidative physiology in either sex. These results suggest that immune strength in males is reduced during breeding, potentially due to resource trade‐offs. Females, on the other hand, appear to be better able to minimise the physiological costs of breeding. Our results suggest that despite the apparent differences between sexes in the effects of sexual selection and parental effort on measures of the two physiological systems, sex differences in physiology cannot be explained by sex roles alone (Vincze et al. 2022). Therefore, the development of sex differences in physiology is most likely influenced by other life‐history and ecological traits, which remain to be explored by future studies. Our results can possibly explain the male‐biased mortality pattern observed in birds (Xirocostas et al. 2020) and are in line with the interspecific variation in avian mortality bias driven by males, specifically via the costs of both mating competition and parental care (Liker and Székely 2005).

## Author Contributions

**Péter L. Pap:** conceptualization (equal), data curation (equal), formal analysis (supporting), funding acquisition (lead), methodology (supporting), project administration (equal), resources (equal), writing – original draft (equal). **Csongor I. Vágási:** conceptualization (equal), data curation (equal), formal analysis (supporting), funding acquisition (supporting), methodology (equal), project administration (supporting), resources (equal), writing – original draft (equal). **Veronika Bókony:** formal analysis (equal), writing – original draft (supporting). **Janka Pénzes:** methodology (equal). **Krisztián Szabó:** methodology (equal). **Nóra M. Magonyi:** methodology (equal). **Gábor Á. Czirják:** methodology (equal). **Orsolya Vincze:** conceptualization (equal), data curation (equal), formal analysis (lead), funding acquisition (supporting), methodology (supporting), project administration (supporting), writing – original draft (equal).

## Conflicts of Interest

The authors declare no conflicts of interest.

## Supporting information


Table S1

Table S2

## Data Availability

The data set used for this study will be freely available for download from the Dryad data repository. For review purposes, the data are provided as the data will be available from the Dryad and will be deleted as supplementary files.
