# Supplementary material for: Phylogenetic Relationships of Immune Function and Oxidative Physiology With Sexual Selection and Parental Effort in Male and Female Birds
Source: Ecol Evol. 2025 Mar 18;15(3):e71119. doi: 10.1002/ece3.71119 (PMC11919744; doi:10.1002/ece3.71119)
Supplement: Supplementary file 1 — Table S1 [file ECE3-15-e71119-s002.doc]

**Table S1.** Phylogenetic generalized least squares models explaining the variation in immune variables between altricial species in relation to polygyny score, sexual dichromatism, parental effort, food type and body mass in males (A) and females (B). Each column represents a separate multivariate model with the variable indicated in the column heading as a response variable. In each cell we report the slope (its standard error in brackets) and *t*-values from PGLS model summaries. Statistical significance of each parameters is coded as * *p* < 0.05, ** *p* < 0.01, *** *p* < 0.001 (highlighted in bold), while marginally significant *p*-values (0.05 < *p* < 0.1) are marked with ^§^. The intercept refers to species with animal-based diets and zero values for the other predictors; all other parameter estimates express slopes or differences from the intercept. Model statistics are reported in the last line of the table, including the strength of phylogenetic signal (Pagel’s λ) and the degrees of freedom (d.f., overall and residual) for each model.

A. Male

|  | **White blood cells**  *β* (SE)  *t*-value | **Heterophils**  *β* (SE)  *t*-value | **Lymphocytes**  *β* (SE)  *t*-value | **H:L ratio**  *β* (SE)  *t*-value | **Agglutination**  *β* (SE)  *t*-value | **Lysis**  *β* (SE)  *t*-value | **Bacteria killing activity**  *β* (SE)  *t*-value |
| --- | --- | --- | --- | --- | --- | --- | --- |
| Intercept | **–0.84 (0.16)**  **–5.43***** | **–0.74 (0.12)**  **–2.33*** | **–0.51 (0.15)**  **–3.30**** | –0.16 (0.17)  –0.94 | –0.26 (0.24)  –1.07 | –0.29 (0.27)  –1.08 | 0.43 (0.88)  0.49 |
| Polygyny score | –0.04 (0.03)  –1.48 | –0.02 (0.02)  –0.69 | –0.04 (0.03)  –1.68^§^ | 0.01 (0.03)  0.35 | –0.05 (0.04)  –1.30 | 0.00 (0.05)  –0.11 | –0.15 (0.14)  –1.09 |
| Sexual dichromatism | **–0.06 (0.03)**  **–2.01*** | **–0.05 (0.02)**  **–2.14*** | –0.04 (0.03)  –1.48 | –0.01 (0.03)  –0.31 | 0.03 (0.04)  0.80 | 0.04 (0.05)  0.74 | 0.03 (0.15)  0.22 |
| Parental effort | 0.00 (0.02)  0.21 | 0.00 (0.01)  –0.37 | 0.01 (0.01)  0.41 | 0.00 (0.02)  –0.16 | –0.04 (0.02)  –1.94^§^ | **–0.07 (0.02)**  **–3.02**** | –0.21 (0.08)  **–2.83**** |
| Food  Omnivorous    Herbivorous | 0.11 (0.10)  1.16  –0.09 (0.12)  –0.76 | 0.11 (0.08)  1.35  –0.01 (0.10)  –1.03 | –0.01 (0.09)  –0.09  –0.04 (0.12)  –0.34 | 0.15 (0.10)  1.51  0.00 (0.13)  –0.03 | –0.13 (0.13)  –0.97  **–0.55 (0.17)**  **–3.31**** | –0.25 (0.17)  –1.51  –0.80 (0.21)  **–3.91***** | –0.48 (0.48)  –1.00  **–1.65 (0.63)**  **–2.63*** |
| Male body mass | **0.24 (0.03)**  **7.44***** | **0.22 (0.03)**  **8.62***** | **0.14 (0.03)**  **4.48***** | 0.05 (0.03)  1.56 | **0.15 (0.04)**  **3.46***** | **0.24 (0.06)**  **4.20***** | 0.32 (0.17)  1.83^§^ |
| Pagel’s *λ* (d.f.) | 0.00 (108, 101) | 0.00 (108, 101) | 0.07 (108, 101) | 0.10 (106, 99) | 0.23 (107, 100) | 0.23 (113, 106) | 0.16 (69, 62) |

^a^Contrasts between omnivorous and herbivorous groups: white blood cells 0.21 (0.14), *t* = 1.49, *p* = 0.3004, heterophils 0.21 (0.11), *t* = 1.85, *p* = 0.1618, lymphocytes 0.03 (0.13), *t* = 0.24, *p* = 0.9701, H:L ratio 0.12 (0.13), *t* = 0.93, *p* = 0.6244, agglutination 0.42 (0.19), *t* = 2.27, *p* = 0.0650, lysis 0.55 (0.23), *t* = 2.40, *p* = 0.0549, bacteria killing activity 1.17 (0.69), *t* = 1.69, *p* = 0.2168.

B. Female

|  | **White blood cells**  *β* (SE)  *t*-value | **Heterophils**  *β* (SE)  *t*-value | **Lymphocytes**  *β* (SE)  *t*-value | **H:L ratio**  *β* (SE)  *t*-value | **Agglutination**  *β* (SE)  *t*-value | **Lysis**  *β* (SE)  *t*-value | **Bacteria killing activity**  *β* (SE)  *t*-value |
| --- | --- | --- | --- | --- | --- | --- | --- |
| Intercept | **–0.75 (0.24)**  **–3.13**** | **–0.64 (0.19)**  **–3.39**** | **–0.55 (0.23)**  **–2.38*** | 0.24 (0.21)  1.12 | –0.43 (0.31)  –1.40 | –0.24 (0.46)  –0.51 | 0.61 (1.34)  0.46 |
| Polygyny score | 0.00 (0.03)  0.12 | 0.01 (0.02)  0.49 | 0.02 (0.03)  0.78 | –0.04 (0.03)  –1.38 | 0.01 (0.04)  0.34 | 0.03 (0.05)  0.54 | –0.21 (0.18)  –1.18 |
| Sexual dichromatism | –0.02 (0.03)  –0.62 | –0.03 (0.03)  –1.28 | 0.02 (0.03)  0.55 | **–0.06 (0.02)**  **–2.64*** | –0.01 (0.04)  –0.22 | –0.06 (0.06)  –1.08 | –0.28 (0.20)  –1.42 |
| Parental effort | 0.00 (0.02)  –0.27 | 0.00 (0.01)  0.06 | 0.00 (0.02)  0.06 | –0.02 (0.01)  –1.51 | –0.01 (0.02)  –0.74 | –0.01 (0.03)  –0.44 | –0.08 (0.09)  –0.94 |
| Food  Omnivorous  Herbivorous | –0.22 (0.11)  –1.96^§^  0.02 (0.12)  0.18 | –0.13 (0.09)  –1.46  –0.01 (0.10)  –0.08 | –0.16 (0.11)  –1.49  0.02 (0.12)  0.13 | 0.14 (0.10)  1.32  0.06 (0.10)  0.63 | –0.10 (0.13)  –0.79  0.04 (0.16)  0.27 | 0.10 (0.19)  0.55  –0.25 (0.24)  –1.04 | 0.77 (0.66)  1.17  0.31 (0.73)  0.43 |
| Female body mass | **0.26 (0.03)**  **7.80***** | **0.21 (0.03)**  **7.97***** | **0.16 (0.03)**  **4.91***** | 0.00 (0.02)  0.18 | **0.18 (0.04)**  **4.28***** | **0.16 (0.06)**  **2.70**** | 0.29 (0.21)  1.36 |
| Pagel’s *λ* (d.f.) | 0.00 (99, 92) | 0.00 (99, 92) | 0.00 (99, 92) | 0.80 (93, 86) | 0.32 (96, 89) | 0.44 (96, 89) | 0.31 (55, 47) |

^a^Contrasts between omnivorous and herbivorous groups: white blood cells –0.24 (0.15), *t* = –1.57, *p* = 0.2663, heterophils –0.12 (0.12), *t* = –0.99, *p* = 0.5843, lymphocytes –0.12 (0.12), *t* = –0.99, *p* = 0.5873, H:L ratio 0.07 (0.13), *t* = 0.56, *p* = 0.8439, agglutination –0.15 (0.20), *t* = –0.75, *p* = 0.7376, lysis 0.35 (0.29), *t* = 1.23, *p* = 0.4407, bacteria killing activity 0.46 (0.87), *t* = 0.53, *p* = 0.8590.
