# Supplementary material for: Phylogenetic Relationships of Immune Function and Oxidative Physiology With Sexual Selection and Parental Effort in Male and Female Birds
Source: Ecol Evol. 2025 Mar 18;15(3):e71119. doi: 10.1002/ece3.71119 (PMC11919744; doi:10.1002/ece3.71119)
Supplement: Supplementary file 2 — Table S2 [file ECE3-15-e71119-s001.doc]

**Table S2.** Phylogenetic generalized least squares models explaining the variation in oxidative physiology measures between altricial species in relation to polygyny score, sexual dichromatism, parental effort, food type and body mass in males (A) and females (B). Each column represents a separate multivariate model with the variable indicated in the column heading as a response variable. In each cell we report the slope (its standard error in brackets) and *t*-values from PGLS model summaries. Statistical significance of each parameters is coded as * *p* < 0.05, ** *p* < 0.01, *** *p* < 0.001 (highlighted in bold), while marginally significant *p*-values (0.05 < *p* < 0.1) are marked with ^§^. The intercept refers to species with animal-based diets and zero values for the other predictors; all other parameter estimates express slopes or differences from the intercept. Model statistics are reported in the last line of the table, including the strength of phylogenetic signal (Pagel’s λ) and the degrees of freedom (d.f., overall and residual) for each model.A. Male

|  | **Total antioxidant status**  β (SE) t-value | **Uric acid**  β (SE) t-value | **Total glutathione**  β (SE) t-value | **Reactive oxygen metabolites**  β (SE) t-value | **Malondialdehyde**  β (SE) t-value |
| --- | --- | --- | --- | --- | --- |
| Intercept | 0.02 (0.01), 1.64^§^ | 0.26 (0.19), 1.33 | **–0.36 (0.13), –2.82**** | 0.02 (0.04), 0.57 | **0.16 (0.07), 2.19*** |
| Polygyny score | –0.00 (0.00), –0.48 | 0.03 (0.03), 0.91 | 0.04 (0.02), 1.95^0.06^ | 0.00 (0.01), 0.34 | 0.00 (0.01), –0.13 |
| Sexual dichromatism | 0.00 (0.00), 0.30 | 0.00 (0.04), 0.01 | 0.01 (0.02), 0.23 | 0.01 (0.01), 1.61 | 0.00 (0.01), –0.24 |
| Parental effort | –0.00 (0.00), –1.44 | –0.01 (0.02), –0.28 | 0.00 (0.01), 0.10 | 0.01 (0.00), 1.78 | –0.01 (0.01), –0.92 |
| Food  Omnivorous  Herbivorous | –0.02 (0.01), –1.84^§^  0.00 (0.01), 0.09 | –0.24 (0.13), –1.83^§^  **–0.39 (0.15), –2.57*** | 0.11 (0.08), 1.36  **0.23 (0.10), 2.35*** | 0.03 (0.02), 1.75^§^  **0.06 (0.03), 2.14*** | **–0.13 (0.04), –3.22****  **–0.15 (0.05), –2.96**** |
| Male body mass | –0.00 (0.00), –0.66 | –0.05 (0.04), –1.27 | **0.08 (0.03), 2.91**** | **–0.03 (0.01), –3.14**** | –0.02 (0.01), –1.83^§^ |
| Pagel’s *λ* (d.f.) | 0.00 (101, 94) | 0.00 (102, 95) | 0.06 (100, 93) | 0.18 (64, 57) | 0.35 (101, 94) |

^a^Contrasts between omnivorous and herbivorous groups: total antioxidant status −0.02 (0.01), *t* = −1.44, *p* = 0.3259, uric acid 0.15 (0.18), *t* = 0.87, *p* = 0.6634, total glutathione −0.12 (0.11), *t* = −1.06, *p* = 0.5419, reactive oxygen metabolites −0.02 (0.03), *t* = −0.80, *p* = 0.7055, malondialdehyde 0.02 (0.06), *t* = 0.37, *p* = 0.9280.

B. Female

|  | **Total antioxidant status**  β (SE) t-value | **Uric acid**  β (SE) t-value | **Total glutathione**  β (SE) t-value | **Reactive oxygen metabolites**  β (SE) t-value | **Malondialdehyde**  β (SE) t-value |
| --- | --- | --- | --- | --- | --- |
| Intercept | **0.03 (0.01), 2.24*** | 0.50 (0.25), 1.98^§^ | –0.13 (0.19), –0.68 | **–0.08 (0.03), –2.68^**^** | 0.13 (0.09), 1.47 |
| Polygyny score | –0.00 (0.00), –1.80^§^ | –0.06 (0.03), –1.90^§^ | 0.01 (0.03), 0.49 | 0.01 (0.00), 1.67 | –0.00 (0.01), –0.36 |
| Sexual dichromatism | –0.00 (0.00), –0.71 | 0.01 (0.03), 0.30 | 0.03 (0.03), 1.01 | **0.01 (0.00), 3.02**** | 0.00 (0.01), 0.13 |
| Parental effort | –0.00 (0.00), –0.57 | –0.00 (0.02), –0.10 | –0.02 (0.01), –1.30 | 0.00 (0.00), 1.56 | –0.00 (0.01), –0.06 |
| Food  Omnivorous  Herbivorous | –0.00 (0.01), –0.66  –0.01 (0.01), –1.72^§^ | –0.00 (0.12), –0.02  **–0.34 (0.14), –2.48*** | –0.07 (0.09), –0.75  0.10 (0.10), 0.98 | 0.00 (0.02), –0.19  –0.02 (0.02), –1.29 | –0.05 (0.04), –1.34  **–0.15 (0.04), –3.33**** |
| Female body mass | **–0.00 (0.00), –2.49*** | **–0.11 (0.04), –3.08**** | **0.07 (0.03), 2.62*** | 0.01 (0.01), 1.05 | –0.02 (0.01), –1.83^§^ |
| Pagel’s *λ* (d.f.) | 0.00 (87, 80) | 0.09 (89, 82) | 0.00 (90, 83) | 0.18 (58, 51) | 0.11 (90, 83) |

^a^Contrasts between omnivorous and herbivorous groups: total antioxidant status 0.01 (0.01), *t* = 0.92, *p* = 0.6322, uric acid 0.34 (0.17), *t* = 2.01, *p* = 0.1245, total glutathione −0.16 (0.12), *t* = −1.35, *p* = 0.3715, reactive oxygen metabolites 0.02 (0.02), *t* = 0.95, *p* = 0.6131, malondialdehyde 0.09 (0.05), *t* = 1.77, *p* = 0.1891.
